# Transcriptional profile of maize roots under acid soil growth

**DOI:** 10.1186/1471-2229-10-196

**Published:** 2010-09-09

**Authors:** Lucia Mattiello, Matias Kirst, Felipe R da Silva, Renato A Jorge, Marcelo Menossi

**Affiliations:** 1 Laboratório de Genoma Funcional, Departamento de Genética, Evolução e Bioagentes, Instituto de Biologia, Universidade Estadual de Campinas, Campinas, SP, Brazil; 2 School of Forest Resources and Conservation, Plant Molecular and Cellular Biology Program, Genetics Institute, University of Florida, Gainesville, USA; 3 Empresa Brasileira de Pesquisa Agropecuária, Centro Nacional de Pesquisa de Recursos Genéticos e Biotecnologia, Brasília, DF, Brazil; 4 Departamento de Fisico-Química, Instituto de Química, Universidade Estadual de Campinas, Campinas, SP, Brazil

## Abstract

**Background:**

Aluminum (Al) toxicity is one of the most important yield-limiting factors of many crops worldwide. The primary symptom of Al toxicity syndrome is the inhibition of root growth leading to poor water and nutrient absorption. Al tolerance has been extensively studied using hydroponic experiments. However, unlike soil conditions, this method does not address all of the components that are necessary for proper root growth and development. In the present study, we grew two maize genotypes with contrasting tolerance to Al in soil containing toxic levels of Al and then compared their transcriptomic responses.

**Results:**

When grown in acid soil containing toxic levels of Al, the Al-sensitive genotype (S1587-17) showed greater root growth inhibition, more Al accumulation and more callose deposition in root tips than did the tolerant genotype (Cat100-6). Transcriptome profiling showed a higher number of genes differentially expressed in S1587-17 grown in acid soil, probably due to secondary effects of Al toxicity. Genes involved in the biosynthesis of organic acids, which are frequently associated with an Al tolerance response, were not differentially regulated in both genotypes after acid soil exposure. However, genes related to the biosynthesis of auxin, ethylene and lignin were up-regulated in the Al-sensitive genotype, indicating that these pathways might be associated with root growth inhibition. By comparing the two maize lines, we were able to discover genes up-regulated only in the Al-tolerant line that also presented higher absolute levels than those observed in the Al-sensitive line. These genes encoded a lipase hydrolase, a retinol dehydrogenase, a glycine-rich protein, a member of the WRKY transcriptional family and two unknown proteins.

**Conclusions:**

This work provides the first characterization of the physiological and transcriptional responses of maize roots when grown in acid soil containing toxic levels of Al. The transcriptome profiles highlighted several pathways that are related to Al toxicity and tolerance during growth in acid soil. We found several genes that were not found in previous studies using hydroponic experiments, increasing our understanding of plant responses to acid soil. The use of two germplasms with markedly different Al tolerances allowed the identification of genes that are a valuable tool for assessing the mechanisms of Al tolerance in maize in acid soil.

## Background

Acid soils are the most important cause of low yield for many crops [1]. About 30% of the world's soils are acidic, and 60% of them are in tropical and subtropical areas associated with long periods of hot and moist weather [1]. Soil acidification is an increasing problem in the United States and Europe because of acid rain, removal of natural plant coverage from large production areas and the use of ammonium-based fertilizers [2]. One of the major problems caused by soil acidification is aluminum (Al) phytotoxicity. Al is the principal component of mineral soils and is present in a wide range of primary and secondary minerals [3]. In soils with pH above 5, Al is precipitated predominately in gibsit form (Al(OH)_3_) and has no phytotoxic effect. At lower pH, Al(OH)_3 _is solubilized and Al is released.

The most evident symptom of Al toxicity is the inhibition of root growth. In maize root tips, Al induces a rapid change in cell number and positioning [4], and recent evidence suggests that DNA damage and interference with cell-cycle progression and cell differentiation are the primary causes of root growth inhibition due to Al toxicity [5]. Other reported effects of Al exposure are the disruption of Ca^2+ ^homeostasis [6,7], increased ACC oxidase activity with a consequent increase in ethylene production [8], Al binding to cell wall polysaccharides [9,10] and reduced membrane fluidity [11].

To cope with Al stress, plants activate exclusion and tolerance mechanisms [1]. Exclusion mechanisms take place outside the roots and prevent the entry of Al into the cell. These mechanisms include cell wall Al immobilization, increased selective permeability of the plasma membrane, rhizosphere pH barrier formation and quelling by exudates such as organic acids and phenolic compounds [1,12-15]. Tolerance mechanisms are active after Al enters the cell - Al ions can be quelled in the cytosol, compartmentalized inside the vacuole or proteins that bind directly to Al may become highly expressed [12,16,17].

Among all of the proposed mechanisms, organic acid release is the most well-characterized resistance strategy used by plants. Since the first report demonstrating Al-induced malate secretion in wheat [18], several research groups have observed that organic acid exudation is higher in tolerant than sensitive genotypes in species such as snap beans [19], wheat [20] and maize [21-24]. However, in maize and wheat, organic acid release does not correlate with resistance in all genotypes, indicating that other mechanisms, such as active Al exclusion, may also play a relevant role [25-27]. Similarly, Maron et al. [28] and Kumari et al. [29] recently demonstrated that tolerance in maize and Arabidopsis is not associated with increased expression of genes encoding enzymes responsible for organic acid biosynthesis, but rather with differential expression of their transporters.

The identification of genes related to Al tolerance has indicated that a plethora of biological functions are influenced by this ion. With the advent of cDNA arrays, the evaluation of global gene expression changes in response to Al stress allowed the identification of a broader number of genes that are modulated by this ion [28-34]. Guo and colleagues [34] used isogenic lines of wheat with differential tolerance to Al and identified 28 differentially expressed genes, including malate transporters, a β-glucosidase, lectin and a histidine kinase. Kumari et al. [29] reported that exposure to Al induces several ribosomal protein genes, peptidases and phosphatases. Maron et al. [28] compared gene expression in two maize genotypes with contrasting Al tolerance and found that several genes involved in processes such as cell wall remodeling, response to oxidative stress and Pi starvation were differentially regulated.

While the identification of genes related to Al stress has led to a greater understanding of plant responses to this ion, these studies have been conducted mostly using hydroponic culture. This growth condition may not adequately mimic the soil environment with respect to rhizosphere development [35,36], which involves a complex mixture of microorganisms, border cells and mucilage [36]. Several other studies have addressed the role of mucilage in the detection and avoidance of Al toxicity. Horst et al. [37] demonstrated that 50% of all Al in root apexes of *Vigna unguiculata *is sequestered by the mucilage layer, and its removal increases root sensitivity to Al [37]. Similarly, Archambauldt et al. [38] found that mucilage production by sensitive varieties of wheat was inhibited more rapidly than that by tolerant varieties when exposed to phytotoxic levels of Al [38]. Similarly, Miyasaka and Hawes [36] found evidence that in snap beans, border cells are involved in detecting and avoiding Al toxicity. By contrast, Li et al. [39] observed that root growth inhibition in maize was not affected by the removal of root mucilage. These findings indicate that different species, or even different varieties of the same species, can present distinct resistance and/or tolerance mechanisms. Therefore, evaluating Al tolerance in conditions that are similar to those in the field may provide a better understanding of the mechanisms required to avoid Al toxicity.

Here we present an analysis of transcriptome changes in two maize varieties with contrasting levels of Al tolerance, using acid soil as the growth substrate. Our analysis identified genes in several metabolic pathways whose expression was modified when plants were growth in acid soil. While we found some Al-responsive genes previously identified in studies carried out in hydroponic growth conditions, growth in acid soil clearly also triggered a new suite of physiological and transcriptional responses not previously reported. Taken together, our results offer a more complete picture of the transcriptomic changes imposed by acid soils, and they may lead to the discovery of novel genes involved in Al tolerance.

## Results

### Physiology of maize seedlings grown in acid soil

**Figure 1:**
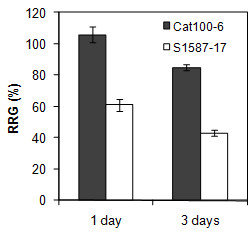
**Effect of acid soil saturated with Al^3+ ^on maize root growth**. Plants were grown in acid (pH 4.2) or control (pH 5.5) soil for one or three days. The growth is relative to the control soil (pH 5.5). Vertical error bars represent mean ± SE (n = 20). The difference between the two lines in each treatment was significant at *p *< 0.05.

**Figure 2:**
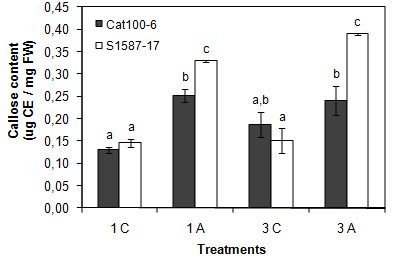
**Acid soil-induced callose deposition in root tips**. Each bar represents the callose content of root apexes grown on acid soil or control soil. C: control soil; A: acid soil; one or three days of treatment. Each quantification refers to the mean ± SD (n = 20). The experiment was done in duplicate. Means with different letters are significantly different (*p *< 0.05) from each other.

**Figure 3:**
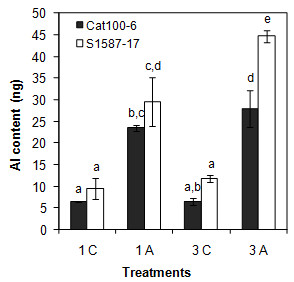
**Al^3+ ^quantification in soil grown maize seedlings**. The experiment was done using the first 5 mm of 10 root tips. C: control soil; A: acid soil; one or three days of treatment. Bars refer to the mean ± SD of the Al content of 10 root tips (n = 2). The means with different letters are significantly different (p < 0.05) from each other.

Most recent studies aiming to characterize plant transcriptomic or proteomic responses to Al exposure have used hydroponic culture [28,29,40]. In the present study, soil was used as the substrate to better mimic field conditions and to allow the maintenance of all root apex components and root architecture. Plants were grown in Dark Red Latossol (pH 4.1) with an Al content of 10 mmol_c_/dm^3^. As a control, the same soil was used, but with pH corrected to 5.5 (see Methods section). Two lines used in previous studies that evaluated Al tolerance in hydroponic growth conditions were characterized: Cat100-6 (Al-tolerant) and S1587-17 (Al-sensitive) [25,28,33,41-44]. S1587-17 is a somaclonal variant regenerated from a callus culture of Cat100-6 [42]. Relative root growth (RRG) was measured after one and three days of growth in soil. The Al-sensitive plants were severely affected by acid soil at both time points, while the Al-tolerant plants were affected only after three days and to a significantly lower extent (Figure 1; see also Additional file 1: Figure S1). Both maize genotypes had higher levels of callose when grown in acid soil, a response typically correlated with Al stress [45-47]. However, the increase in callose content was significantly higher in the Al-sensitive line (Figure 2). To investigate whether the root inhibition and callose accumulation were due to Al phytotoxicity associated with acid soil, the Al absorbed by root tips was quantified after one and three days of soil exposure. Figure 3 demonstrates that the sensitive line S1587-17 had significantly higher amounts of Al than the Al-tolerant variety. These results indicate that these maize varieties have different physiological responses to acid soil and that these responses are most likely due to the presence of Al. Nevertheless, we cannot exclude the possibility that pH also contributes to plant responses during acid soil exposure.

### Gene Expression Profiling

**Figure 4:**
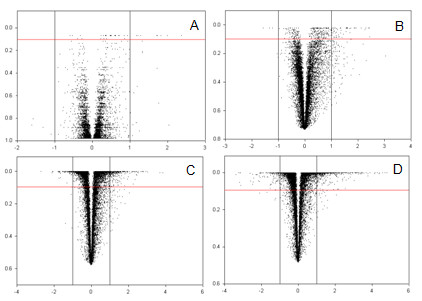
**Volcano plots representing interactions of various effects**. Estimates were calculated as the difference between the least-square means for each comparison (x-axis). Estimates equal to zero represent no expression change and estimates different from zero represent gene expression modifications. A: interaction effect between genotype, treatment and time for Cat100-6 one day; B: interaction effect between genotype, treatment and time Cat100-6 three days; C: interaction effect between genotype, treatment and time S1587-17 one day; D: interaction effect between genotype, treatment and time S1587-17 three days. The red line represents an FDR of 10%, and consequently data points above this line represent significant observations (the y-axis represents Qvalues). Note that the Estimate axis is different for each plot.

**Figure 5:**
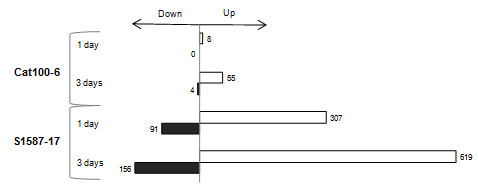
**Diagram representing the number of genes differentially expressed in each genotype at each time point**.

The Affymetrix GeneChip^® ^Maize Genome Array was used to evaluate the transcriptional response of the two contrasting maize genotypes to growth in acid soil. Analysis of variance (ANOVA) was used to dissect the transcriptional responses associated with the individual maize lines (independent of soil treatment or time of exposure), time of collection (1 and 3 days), soil type (control or acid soil treated) and all possible interactions (see Methods section). In the Al-tolerant line (Cat100-6) exposed to acid soil for one day, only eight genes were differentially expressed compared to plants grown in control soil (Figure 4A). The number of differentially regulated genes increased (59) after three days of treatment (Figure 4B). However, the Al-sensitive maize line showed a significantly higher number of differentially expressed genes. On the first day, 339 genes were differentially regulated (Figure 4C), while 776 were affected by the treatment on the third day (Figure 4D). The genes that were differentially regulated under all conditions are described in the Additional file 2 (Tables S1-S4), and the number of genes up- or down-regulated in each genotype and at each time point is shown in Figure 5.

**Figure 6:**
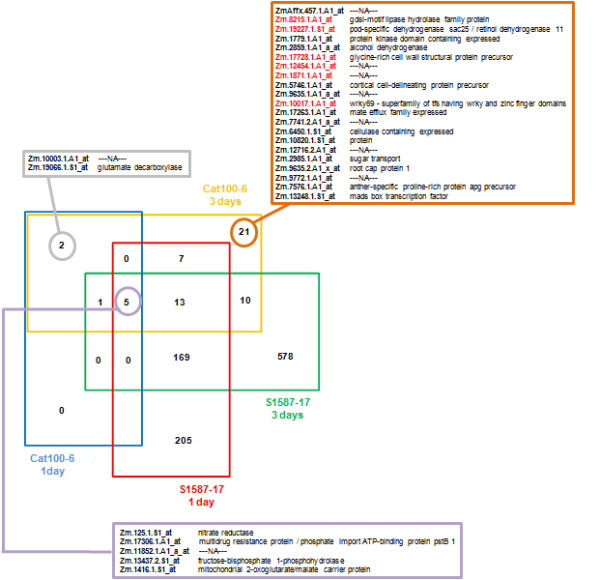
**Multiple comparison Venn diagram**. Each quarter represents a list of IDs of genes differentially expressed in the comparison between acid and control soils for each maize variety. The boxes indicate the Affymetrix ID and the annotation of the genes identified only in Cat100-6 (both time points - grey box), in all genotypes and time points (purple box) and genes identified only in Cat100-6 after three days (orange box - the genes identified in red are those that also presented significantly higher expression values in Cat100-6 in the comparison between genotypes after three days in acid soil).

Figure 6 depicts the number of genes differentially regulated between acid and control soil conditions that are unique or shared between S1587-17 and Cat100-6 at each time point. All of the eight genes differentially regulated on day 1 in Cat100-6 were up-regulated, and they were also detected after three days of acid soil exposure. Only two genes were exclusively detected in the Al-tolerant line: Zm.10003.1.A1_at (no hit) and Zm.19066.1.S1_at (glutamate decarboxylase). Five genes were differentially expressed in both S1587-17 and Cat100-6 at one and three days: Zm.125.1.S1_at (nitrate reductase), Zm.17306.1.A1_at (multidrug resistance protein/phosphate import ATP-binding protein pstB 1), Zm.11852.1.A1_a_at (no hit), Zm.13437.2.S1_at (fructose-bisphosphate 1-phosphohydrolase) and Zm1416.1.S1_at (mitochondrial 2-oxoglutarate/malate carrier protein).

To further evaluate the quantitative extent by which genes were differentially regulated between the two maize lines, the difference in the least-square means estimates (DEs) of gene expression levels between Cat100-6 and S1587-17 grown in acid soil were calculated at each time point (e.g., Cat100-6 in acid soil for three days versus S1587-17 in acid soil for three days), and the statistical significance was assessed. We aimed to identify genes induced by acid soil in Cat100-6 that also had significantly higher absolute levels than in S1587-17. We observed that none of the genes differentially expressed in Cat100-6 after one day of acid soil treatment presented significantly higher expression levels than in S1587-17 under the same conditions. However, eight genes out of the 59 differentially expressed in Cat100-6 after three days were also significantly more highly expressed relative to S1587-17 growing in acid soil for three days. Six of these genes are in the group of 21 exclusively identified as differentially regulated in Cat100-6 after three days (Figure 6), while the other two were also differentially expressed in S1587-17 after three days (Zm.3371.1.A1_at - O-methyltransferase and Zm.8742.1.A1_at - unknown protein).

Due to the large number of genes identified in the Al-sensitive maize, a functional analysis was performed with Gene Ontologies to help identify the pathways affected by the toxicity imposed by acid soil. Most of the genes up-regulated on the first day are involved in lipid metabolism, oxidative stress responses and cell wall metabolism (Additional file 1: Figure S2A). Most of the genes down-regulated on the first day encode proteins involved in DNA packaging and cell cycle (Additional file 1: Figure S2B). Most of the genes up-regulated on the third day are involved in cell wall metabolism, oxidative stress responses and anionic transport (Additional file 1: Figure S3A). On the other hand, most of the repressed genes are involved in protein metabolism (Additional file 1: Figure S3B).

### Validation of gene expression profiles using qPCR

**Figure 7:**
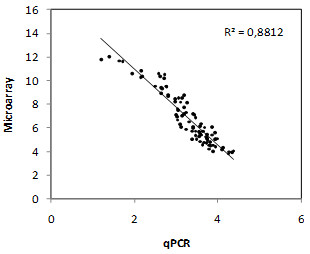
**Real-time qPCR validation of the microarray results**. The qPCR data were log_2 _transformed and plotted against the microarray data (least-square means). The correlation is negative because in the qPCR data, the more the gene is expressed the lower is its Ct value.

To validate the microarray results, eleven differentially expressed genes were selected and real-time qPCR was performed (Figure 7). This validation was done with two independent biological replicates (different from the replicates used for the microarray experiment). A significant correlation between the two data sets was observed (*R^2 ^*= 0.8812).

### Comparison with gene expression responses to Al treatment in hydroponic culture

**Figure 8:**
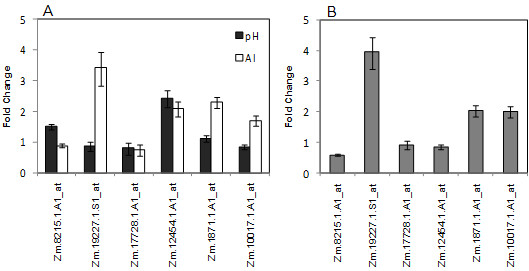
**Expression of selected genes after hydroponic culture**. (A) Data show the relative expression (in fold change) in relation to pH 5.5; (B) Data show the relative expression (in fold change) of the treatment with pH 4.2 plus Al versus pH 4.2 without Al. The results are from three independent biological replicates.

The soil treatment used in this work has two major variables that must be considered, the presence of phytotoxic Al and the pH. However, it is not possible to separate these properties in the soil or even use a different acid soil with no Al because diversity in the physical and chemical characteristics would affect the results. Therefore, hydroponic culture has been used to evaluate the effects of pH and Al levels on the expression of selected genes. Six out of the eight acid soil-induced genes from Cat100-6 that also had absolute levels higher than those of S1587-17 were used in an experiment consisting of three treatments: pH 5.5; pH 4.2 with no Al and pH 4.2 with 36 μM Al. The effect on gene expression in Cat100-6 seedlings is illustrated in Figure 8A (pH-effect, using pH 5.5 as the reference). The relative expression in seedlings grown in pH 4.2 with Al relative to pH 4.2 without Al (Al-effect) is shown in Figure 8B. Genes such as Zm.8215.1.A1_at (GDSL-motif lipase hydrolase family protein), Zm.17728.1.A1_at (glycine-rich cell wall structural protein precursor) and Zm.12454.1.A1_at (protein with unknown function) showed no significant differential regulation under treatment with pH 4.2 in the presence or absence of Al or between pH 4.2 and pH 5.5 treatments, suggesting significant differences between the gene expression profiles from the hydroponic and soil experiments. However, Zm.19227.1.S1_at (Pod-specific dehydrogenase SAC25/retinol dehydrogenase 11), Zm.1871.1.A1_at (protein with unknown function) and Zm.10017.1.A1_at (WRKY 69 transcription factor) were up-regulated in the presence of Al, indicating that Al and not pH was the main factor behind their induction in the soil treatment.

To further highlight the different responses of maize when grown in hydroponics versus soil, we compared the target sequences from the Affymetrix platform used in this work with the target sequences from Maron et al. [28]. We observed that only a fraction of the genes modulated by Al in Cat100-6 grown in the hydroponic experiment were also modulated in acid soil-grown plants: of the 59 genes differentially expressed in Cat100-6 grown in soil, only six were also found in the hydroponic assay. We also compared our data from S1587-17 with the data from the Al-sensitive line L53 because in the work of Maron et al., [28] S1587-17 was not used. In this case, the differences were even higher because only 34 out 952 genes modulated in S1587-17 were common to both gene sets. A complete list of the genes found in both experiments is shown in Additional file 2: Table S5.

## Discussion

### Physiology of maize roots grown in acid soil

Plant tolerance to Al is usually evaluated using hydroponic culture [48-53], but conflicting results may be obtained when compared to soil conditions [54,55]. Even when Al tolerance is consistent between hydroponic and soil conditions [56-60], plant responses may differ in the two growth systems. To address this limitation, we evaluated maize tolerance to acid soil containing 10 mmol_c_/dm^3 ^(equivalent to 90 ppm) of Al and at pH 4.2. These conditions are within the range of previous studies and are sufficient to elicit a phytotoxic response [35,61-63], allowing phenotypic discrimination between two maize lines with distinct levels of resistance to Al, Cat100-6 (tolerant) and S1587-17 (sensitive). As shown in previous studies using these and other maize lines [25,28,39-42,46], Cat100-6 accumulated less Al in its root tips when grown in acid soil when compared to S1587-17. The amount of Al absorbed by root tips is indicative of the sensitivity of plants to this abiotic stress [13,64,65], presumably because genotypes that accumulate less Al in their root apexes have a more efficient exclusion mechanism. However, after prolonged exposure (3 - 5 days), the amount of Al in Cat100-6 roots continued to increase (although at a lower rate than in S1587-17), in disagreement with previous studies that reported a reduction in Al accumulation in root tips after 24 hours in hydroponics [28,41]. Therefore, the exclusion mechanism of Cat100-6 appears to have less activity in soil than in hydroponic conditions. Nonetheless, Al-induced root growth inhibition and callose formation were markedly more limited in Cat100-6 than in S1587-17.

### Gene expression profiles of an Al-tolerant and an Al-sensitive maize line

The abiotic stress caused by the toxicity of acid soil was clearly reflected in the gene expression profiles. The number of genes differentially regulated between control and treated (acid soil) plants increased in both Cat100-6 and S1587-17 when they were exposed to the stress for an extended period. It is worth mentioning that these changes observed in the transcriptome certainly reflect both direct and indirect effects of the stress caused by acid soil. In the field, where roots continuously grow and explore different soil regions, this is also certainly true. Interestingly, a smaller fraction of the transcriptome responded to acid soil stress in the resistant line Cat100-6 than in S1587-17, similarly to previous reports using Al and hydroponics [28,33,66]. Therefore, part of the resistance of Cat100-6 (and Al-tolerant plants generally) may involve a mechanism that limits major disturbances to plant function and thereby avoids a cascade of detrimental downstream effects.

Genes that are modulated by a stress are natural candidates for explaining the defenses activated by plants. By using contrasting genotypes it is possible to narrow the scope to genes with a higher probability of playing major roles in the plant response. Therefore, we selected eight genes that were more highly expressed in Cat100-6 than in S1587-17 under acid soil conditions. Six of these genes were also found to be induced by acid soil only in Cat100-6, while two were also induced in S1587-17, although to a lesser degree. We further characterized those six genes that were specific to Cat100-6, which encoded a GDSL-motif lipase hydrolase family protein, a pod-specific dehydrogenase/retinol dehydrogenase 11, a glycine-rich protein (GRP), a member of the WRKY transcriptional factor family and two unknown proteins. However, the hydroponic experiment demonstrated that only three of these genes have interesting expression patterns (up-regulated during Al treatment - Figure 8). Although further work will be needed to assess the individual contribution of each gene to acid soil tolerance, these data give insight into the strategies used by plants in fields where this stress takes place.

Zm.19227.1.S1_at (Pod-specific dehydrogenase/retinol dehydrogenase 11) was first described as a gene involved in rape oilseed pod development [67]. Members of this family may function as a bridging molecule between the nutritional signaling pathway and the hormone biosynthesis pathway in Arabidopsis [68]. This member is associated with ABA production and is critical for growth and development, and also for plant responses to stress via glucose signaling [68].

The WRKY family of transcription factors was first identified in plants and presents a high number of members [69]. About 70 members have been indentified in Arabidopsis, and several of them are induced in response to wounding, pathogen infection and abiotic stresses such as drought, cold and salinity [31,69-71]. Our microarray experiment indentified Zm.10017.1.A1_at (WRKY69) as differentially expressed in Cat100-6 after three days of acid soil treatment and also as presenting higher expression than in the Al-sensitive S1587-17 genotype. Hydroponic gene expression experiments also demonstrated that Al induces the expression of this gene. Kumari and colleagues [29] identified two WRKY family members as being down-regulated after exposure of Arabidopsis to Al. Meanwhile, Goodwin and Sutter [72], also studying Arabidopsis, identified WRKY 33 as up-regulated due to Al treatment. An additional study identified another WRKY member as up-regulated due to Al and Cd stress. Using the same tolerant variety (Cat100-6) Maron et al. [28] identified two WRKY family members after 6 h of Al treatment. These results suggest that this transcription factor may be involved in the regulation of other genes that contribute to acid soil tolerance in plants.

Zm.1871.1.A1_at is a protein with unknown function that was up-regulated in acid soil and in hydroponics, indicating that Al rather than low pH is the inducer. Interestingly, this protein has a conserved domain typical of methyltransferases (MTase), which are responsible for methylation of several cellular components such as DNA, RNA, proteins and also small molecules [73]. These enzymes may also play important roles in disease resistance, growth and development [74]. Studies with rice [75], Arabidopsis [76] and tomato [40] have also identified members of this family as up-regulated after Al treatment. This is the first study to detect a potential role in acid soil tolerance for this maize protein.

A comparison of the transcriptional profile of roots grown in soil (this work) with that of roots grown hydroponically [28] showed only a minor overlap between these two growth systems. Although such comparisons are difficult because of differences in the chip platforms, conditions in different laboratories and other aspects, it strongly suggest that root responses in acid soil differ at least in part from those observed in hydroponic experiments. However, several pathways that are affected by Al in hydroponics were also observed in acid soil grown plants, as discussed below.

### Organic acid biosynthesis

Of particular interest are genes involved in organic acid biosynthesis, which can protect the plant from deleterious effects of Al by binding to it after being secreted by root apexes [77]. However, only one gene belonging to an organic acid biosynthesis pathway was identified as down-regulated in S1587-17 after a three-day treatment (citrate synthase - Zm.15069.1.A1_at). Previous studies have suggested that Cat100-6 activates pre-existing anionic channels after exposure to Al but prior to activation of the organic acid biosynthesis pathways [23,25]. It has also been observed that the levels of citrate exudation induced by Al in Cat100-6 roots is higher than in other Al-sensitive genotypes (such as L53), but it stays constant over time [28], although no correlation between organic acid exudation and Al-alteration of genes of the organic acid biosynthetic pathway has been observed in maize [28,33].

### Oxidative stress in soil grown plants

Plant cells normally produce reactive oxygen species (ROS) due to cellular processes that result in reduction of oxygen molecules. Plants have several enzymes capable of producing ROS and others that fight ROS to avoid cellular damage. Al toxicity can lead to an imbalance that results in oxidative stress and increases in the activity of enzymes and genes that reduce ROS effects, as previously observed in maize [28,33,41,78,79] and other plant species [80-84]. Specifically in the case of the two maize lines used in this work, a previous study indicates that S1587-17 produces higher levels of ROS under Al stress, while ROS production remains constant in Cat100-6 [41]. Expectedly, genes involved in ROS production such as an oxalate oxidase (Zm.503.1.A1_at) and four germins (Zm.1315.1.A1_at; Zm.2525.1.A1_at; Zm.842.1.A1_at and Zm.9049.1.A1_x_at) were up-regulated in S1587-17 after acid soil treatment. In contrast, the up-regulation of genes implicated in the production of ROS was not detected in the Al-tolerant genotype Cat100-6 under stress.

Higher expression of oxalate oxidases in S1587-17 was also correlated with the up-regulation of peroxidases in the Al-sensitive genotype. A gene encoding a glutathione peroxidase (Zm.6103.1.A1_a_at) was up-regulated in the Al-sensitive line, confirming the data obtained by Boscolo et al. [41], who found higher levels of this enzyme in S1787-17 under Al stress. In fact, more ROS scavenging genes were differentially expressed in S1587-17 than in Cat100-6, possibly reflecting a response to the up-regulation of ROS-producing genes. These data are in agreement with those from Maron et al. [28] suggesting that Cat100-6 has mechanisms that act before the oxidative stress takes place. However, none of the genes encoding superoxide dismutase were identified as up-regulated in S1787-17, in contrast to the induction of this enzyme [41] and transcriptional up-regulation detected previously in hydroponics [28].

The degree of ROS production and the enzymes involved in their metabolism may partially explain the differences in root growth detected in S1587-17 and Cat100-6 in acid soil. Together with oxalate oxidase, peroxidases act to remodel the cell wall during development and stress responses [85,86]. Our results indicate that the elevated number of peroxidases up-regulated in S1587-17 may be one of the causes of the root inhibition in this genotype, either by increasing ROS production or by changing the cell wall structure. On the other hand, Cat100-6 appears to be more effective at preventing ROS generation. This is supported by the smaller number of genes contributing to ROS production compared to the number observed in hydroponic culture [28,33] and also by the constitutive (i.e., independent of soil type) expression of ROS scavenging genes (such as GST) in Cat100-6.

### The phenylpropanoid pathway is activated in Al-sensitive maize plants

The higher expression of peroxidases in S171587-17 in acid soil was also correlated with an increase in the expression of several genes implicated in the synthesis of monolignols. Genes related to lignin biosynthesis have often been identified as responding to Al stress in monocots [28,33,87,88], and higher lignin deposition has been associated with root growth inhibition in Al-sensitive wheat genotypes [87]. The phenylpropanoid pathway is the last biochemical step in the production of monolignols and the lignin polymer. The up-regulation of genes in the shikimate pathway, including shikimate kinases (Zm.3954.1.A1_at and Zm.10310.1.A1_at, are up-regulated in S1587-17/3 days) and chorismate mutase (up-regulated in S1587-17, Zm.9783.1.A1_at and Zm.10652.1.S1_at, and Zm.9867.1.A1_at constitutively expressed in Cat100-6) might increase the production of phenylalanine, the precursor for the phenylpropanoid pathway. Cinnamoyl-reductase (Zm.3297.1.A1_at) and several O-methyltransferases were up-regulated at days 1 and 3 in the Al-sensitive line, indicating that S1587-17 might accumulate lignin, reducing root growth. Similarly, several genes related to callose biosynthesis were up-regulated in the Al-sensitive line at both time points (Zm.16347.1.A1_at - beta-glucan binding protein; Zm.14573.1.S1_at - glucan endo-beta-glucosidase 7 precursor; Zm.5768.1.A1_at - beta-glucanase precursor and Zm.12098.1.A1_at - endo-1,3;1,4-beta-d-glucanase precursor), corroborating the physiological data that show higher levels of callose in S1587-17.

### Insights into hormonal responses to acid soil

Al has also been shown to impact root growth by modifying the levels of phytohormones such as auxin [89] and ethylene [8]. Genes encoding enzymes involved in auxin biosynthesis such as IAA amidohydrolase (Zm.3056.1.A1_at) and anthranilate phosphoribosyltransferase (Zm.1556.1.A1_at) were up-regulated in the root apex of the sensitive line S1587-17 when under acid soil stress, while the auxin-degrading enzyme indole-3-acetate beta-glucosyltransferase (Zm.18805.1.A1_at) was down-regulated after three days of acid soil exposure. Although auxin can induce new root formation, higher concentrations inhibit root elongation and enhance adventitious root formation. The genotype S1587-17 grown in acid soil developed significantly more lateral roots compared to plants grown under control conditions (data not shown). In coordination with the up-regulation of auxin-responsive genes (Zm.16990.1.S1_at, Zm.255.1.A1_at and Zm.5214.1.S1_at), its F-box receptor [90] was also up-regulated in S1587-17 (Zm.15393.1.S1_at). This might indicate a compensatory mechanism of primary root inhibition and lateral root stimulation to avoid nutrient and water deficiencies.

The transcriptome response of the Al-susceptible line S1587-17 to acid soil treatment also involved the up-regulation of two ACC oxidases (Zm.18900.1.S1_at and Zm.7909.1.A1_at), suggesting activation of the ethylene production pathway. The phytohormone ethylene mediates root growth inhibition [91,92], and treatment with inhibitors of ethylene perception increases root elongation [93]. Therefore, increased ethylene production is involved in root growth inhibition in the Al-sensitive genotype, and this response might be modulated not only by the germplasm but also by the culture conditions.

## Conclusion

In this study we have characterized the transcriptomic changes of maize roots growing in acid soil containing toxic levels of Al. Our data highlighted several metabolic pathways that are challenged due to the stress caused by acid soil, including those involved with ROS production and detoxification, cell wall structure and hormone biosynthesis. Several genes previously reported as up-regulated by Al treatment in hydroponic experiments were also identified in acid soil grown plants. Most interestingly, we found genes that provide interesting clues to the mechanisms underlying the acid soil tolerance of an Al-tolerant maize line. These genes encode a GDSL-motif lipase hydrolase family protein, a pod-specific dehydrogenase/retinol dehydrogenase 11, GRP, WRKY and two proteins of unknown function. Taken together, these data provide a better understanding of the basis of Al toxicity and tolerance in acid soils.

## Methods

### Plant material and growth conditions

Seeds from the tropical maize (*Zea mays *L.) inbred lines Cat100-6 and S1587-17 were geminated for two days in moist filter paper. Seedlings with similar initial root length were transferred to 0.5-liter plastic pots with 1 kg of soil (with 15% water - mL/Kg). Each bag received 20 seedlings, which were grown in a growth chamber at 26°C (light: dark, 16:8 h). Bags were weighed twice daily and the weight was completed with distilled water to maintain the humidity at 15%.

Plants were grown in a Dark Red Latossol sieved through a 4-mm mesh. Soil analysis indicted a pH of 4.1 and Al content of 10 mmol_c_/dm^3 ^(referred to as the acid soil treatment). Fertilization was applied to avoid nutritional stress and consisted of the following nutrients (mg/Kg of soil): 56 of N; 38.75 of P; 78 of K; 32 of S; 60 of Mg; 0.5 of B; 0.5 of Cu; 0.01 of Mo; 1.0 of Zn. The soil used in the control treatment was incubated with 0.8 g of Ca(OH)_2 _per Kg for one week prior the fertilization and the same amount of nutrient was added to the acid soil. The incubation with Ca(OH)_2 _increased the pH to pH 5.5 and the presence of free Al was no longer detected. The acid soil also received a correction for Ca through the addition of a CaCl_2 _solution to compensate for the Ca(OH)_2 _added to the control soil. The soil was thoroughly mixed to reduce natural variability of the physical and chemical properties and to ensure homogeneous fertilization.

### Relative root growth (RRG)

Before transferring the seeds to soil, the initial root length of each seedling was measured. After each growth period (1 and 3 days), the pots were cut and the soil was gently removed to expose the roots. Each root was washed in running water to remove the excess soil and the root length was measured. Root growth (RG) was calculated as the final length (after growth in soil) minus the initial length. The relative root growth (RRG) of each maize line was calculated as the RG of all the seedlings grown in acid soil divided by the mean RG of all the seedlings grown in control soil times 100.

### Aluminum quantification

Al quantification was carried out as described by Bloom et al. [94] after the roots were washed in acidified water (pH 4.0). Measurements were performed in a spectrofluorometer (ISS PCI Photon Counting Spectrofluorometer) with lamp intensity of 10 A, emission and excitation gap of 2 mm. The excitation wavelength was 390 nm and the emission wavelength was 497 nm. Each sample was measured 10 times with a quartz cuvette (optical length of 1 cm). A standard curve was made with serial dilutions of AlCl_3_.

### Callose quantification

Callose content was quantified as described by Jones et al. [95] with modifications. Ten root apexes were fixed in formalin. After 48 h, the solution was replaced with 200 μL of NaOH (1 M) and the root tips were disrupted with the use of a pistile. After 24 h, an additional 800 μL of NaOH (1 M) was added to each sample and they were placed in a water bath at 80°C for 15 minutes. The samples were rapidly centrifuged at 1000 g after cooling off. A total of 400 μL of the upper phase was transferred to a new tube and 800 μL of aniline blue solution (0.1% - w/v), 420 μL of HCl (1 M) and 1,180 μL of glycine/NaOH buffer (pH 9.5) were added, and they were incubated at 80°C for 20 minutes. Callose content was quantified in a spectrofluorometer, as described above, but with an excitation wavelength of 385 nm and an emission wavelength of 485 nm. Each sample was read 10 times with a quartz cuvette (optical length of 1 cm). A standard curve was made with serial dilutions of curdlan solution. The amount of Al-induced callose deposition was calculated as the quantity in the acid soil treatment minus the quantity in the control soil treatment.

### RNA extraction

RNA was extracted with an RNeasy Plant Mini Kit (Qiagen, Valencia, USA). The RNA was evaluated in an agarose/formaldehyde gel, quantified in a spectrophotometer and stored at -80°C.

### Microarray hybridization and analysis

For the microarray experiment, three independent replicates were used, for a total of 24 samples. Two micrograms of each RNA sample was processed and hybridized to the Affymetrix GeneChip^® ^Maize Genome Array as described by the manufacturer's protocol. The hybridization, staining, washing and scanning were performed at Laboratório Nacional de Luz Sincrontron (LNLS), Campinas, SP, Brazil, with the use of the Command Console Software (Affymetrix, USA). The data were normalized with the RMA method, log_2 _transformed and loaded into SAS (SAS Institute, USA) to perform the contrasts. A one-way analysis of variance (ANOVA) was used to separate the contribution of each effect on the expression level of a given gene. The model used was: *y*_*ikl *_= μ + *G*_*i *_+ *Ta*_*k *_+ *Te*_*l *_+ *(G *× *Ta)*_*ik *_+ *(G *× *Te)*_*il *_+ *(G *× *Ta *× *Te)*_*ikl *_+ *ξ*_*ikl *_where *μ *is the sample mean, *G*_*i *_represents the genotype effect for the i^th ^genotype (e.g., Cat100-6 or S1587-17) (df = 1), *Ta_k _*is the effect of the k^th ^Treatment (e.g., acid soil or control soil) (df = 1), *Te_l _*is the effect of l^th ^time point (e.g., 1 or 3 days) (df = 1), *(G *× *Ta)*_*ik *_is the effect of interaction between genotype and treatment (df = 1), *(G *× *Te)*_*il *_is the effect of interaction between genotype and time point (df = 1), *(G *× *Ta *× *Te)*_*ikl *_is the effect of interaction between genotype, treatment and time (gl = 1) and *ξ_ikl _*is the residual error. Least-square means for each gene in each sample were generated and differential estimates (DE) of expression were calculated as the difference between least-square means for each of the terms in the model. DE values were calculated between the acid and control soil treatments and also between genotypes. The false discovery rate (FDR) was set to 10% to control Type I errors [96]. Q values were calculated from *P*-values using the software Q-value from the R platform [97]. Only the genes with DE above 1 were further analyzed. The list of differentially expressed genes was annotated with the use of Blast2GO software http://www.blast2go.org[98-100] using default settings.

### Hydroponic culture

To validate the microarray data and to separate the effect of pH from that of Al, a hydroponic experiment was performed. The basic solution consisted of 0.5 mM CaCl_2_; 0.125 mM MgCl_2_; 1 mM KCl; 1 mM NH_4_NO_3_. This basic solution was divided into two portions and their pHs were corrected to 5.5 (control solution) and 4.2. The solution with the pH of 4.2 was again divided in two and one portion received 36 μM AlCl_3_. This Al concentration yielded the same RRG as was obtained with soil treatment (data not shown). The plants were grown in a growth chamber at 26°C (light: dark, 16:8 h) with constant solution aeration.

### Real Time qPCR

To validate the microarray results, RNA from two additional independent replicates was treated with DNase I Amplification Grade (Invitrogen, USA) and cDNA was synthesized from 2 μg of RNA using High Capacity RNA-to-cDNA Kit (Applied Biosystems, USA). Real-time qPCR for eleven genes identified as differentially regulated in at least one of the experimental conditions was performed with an ABI 7500 (Applied Biosystems, USA) using Sybr Green I PCR Master Mix (Applied Biosystems, USA). The primers were designed using Primer Express 2.0 software (Additional file 2: Table S6). The efficiency of each pair was tested with a relative standard curve experiment. The maize tubulin gene (Zm.6045.1.A1_s_at) was used as an endogenous control. As the efficiency of all the primers was near 100%, the relative expression was calculated by the ΔΔCt method. For microarray validation, the ΔCt values were calculated for each gene in each sample, log_2 _transformed and plotted against its corresponding least-square means data from the microarray. For the hydroponic experiment, the ΔΔCt values were calculated relative to the ΔCt from the pH 5.5 treatment.

## Authors' contributions

LM designed the experiments; performed the microarray hybridizations, data analysis and interpretation; and drafted the manuscript. MK evaluated the data from the microarrays and edited the manuscript. FRS helped with the comparison of the data from the acid soil and hydroponic experiments. RAJ helped with the experiments in acid soil and interpretation of the data. MM designed the experimental approach, led and coordinated the project and edited the manuscript. All authors have read and approved the final manuscript.

## Note

**Accession numbers: **The gene expression data were deposited at The Gene Expression Omnibus (GEO) Database under access number GSE21070

**Supplementary Materials: **Submitted as a additional files

## Supplementary Material

Additional file 1 **Figure S1**. Root phenotype under control (first and third row) and acid soil (second and fourth row) conditions after one day of treatment (A), three days of treatment (B). **Figure S2**. Functional analysis of genes differentially expressed in S1587-17 after one day of treatment in acid soil. A: Up-regulated; B: Down-regulated. All of the genes that did not present Gene Ontologies were removed from the analysis. **Figure S3**. Functional analysis of genes differentially expressed in S1587-17 after three days of treatment in acid soil. A: Up-regulated; B: Down-regulated. All of the genes that did not present Gene Ontologies were removed from the analysis. Click here for file

Additional file 2 **Table S1: Genes differentially expressed in Cat100-6 after one day of acid soil treatment**. FDR = 10% and DE ≤ 1. **Table S2**: Genes differentially expressed in Cat100-6 after three days of acid soil treatment. FDR = 10% and DE ≤ 1. **Table S3**: Genes differentially expressed in S1587-17 after one day of acid soil treatment. FDR = 10% and DE ≤ 1. **Table S4**: Genes differentially expressed in S1587-17 after three days of acid soil treatment. FDR = 10% and DE ≤ 1. **Table S5**: Genes that were differentially expressed in Cat100-6 grown both in soil (this work) and hydroponics [27] and in S1587-17 grown in soil (this work) and L53 in hydroponics [27]. **Table S6**: Primers designed for real-time qPCR. Click here for file
