# Supplementary material for: Transcriptional profile of maize roots under acid soil growth
Source: BMC Plant Biol. 2010 Sep 9;10:196. doi: 10.1186/1471-2229-10-196 (PMC2956545; doi:10.1186/1471-2229-10-196)
Supplement: Additional file 1 — Figure S1. Root phenotype under control (first and third row) and acid soil (second and fourth row) conditions after one day of treatment (A), three days of treatment (B). Figure S2. Functional analysis of genes differentially expressed in S1587-17 after one day of treatment in acid soil. A: Up-regulated; B: Down-regulated. All of the genes that did not present Gene Ontologies were removed from the analysis. Figure S3. Functional analysis of genes differentially expressed in S1587-17 after three days of treatment in acid soil. A: Up-regulated; B: Down-regulated. All of the genes that did not present Gene Ontologies were removed from the analysis. [file 1471-2229-10-196-S1.PDF]

Supplemental Material 1

Mattiello et al. (2010) BMC Plant Biology

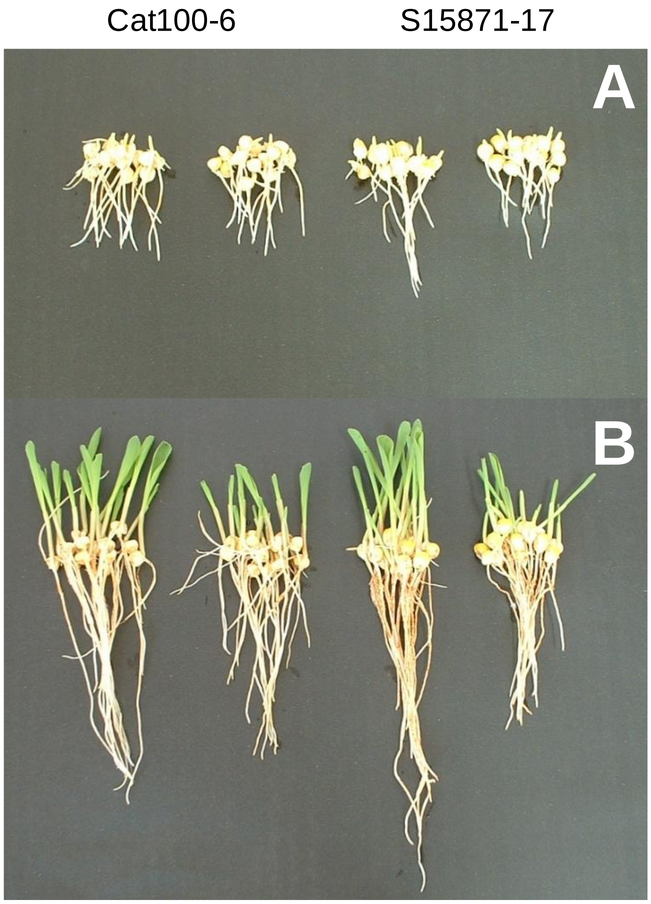

Figure S1. Root phenotype under control (first and third row) and acid soil (second and fourth row) for 1 day treatment (A), 3 days treatment (B).

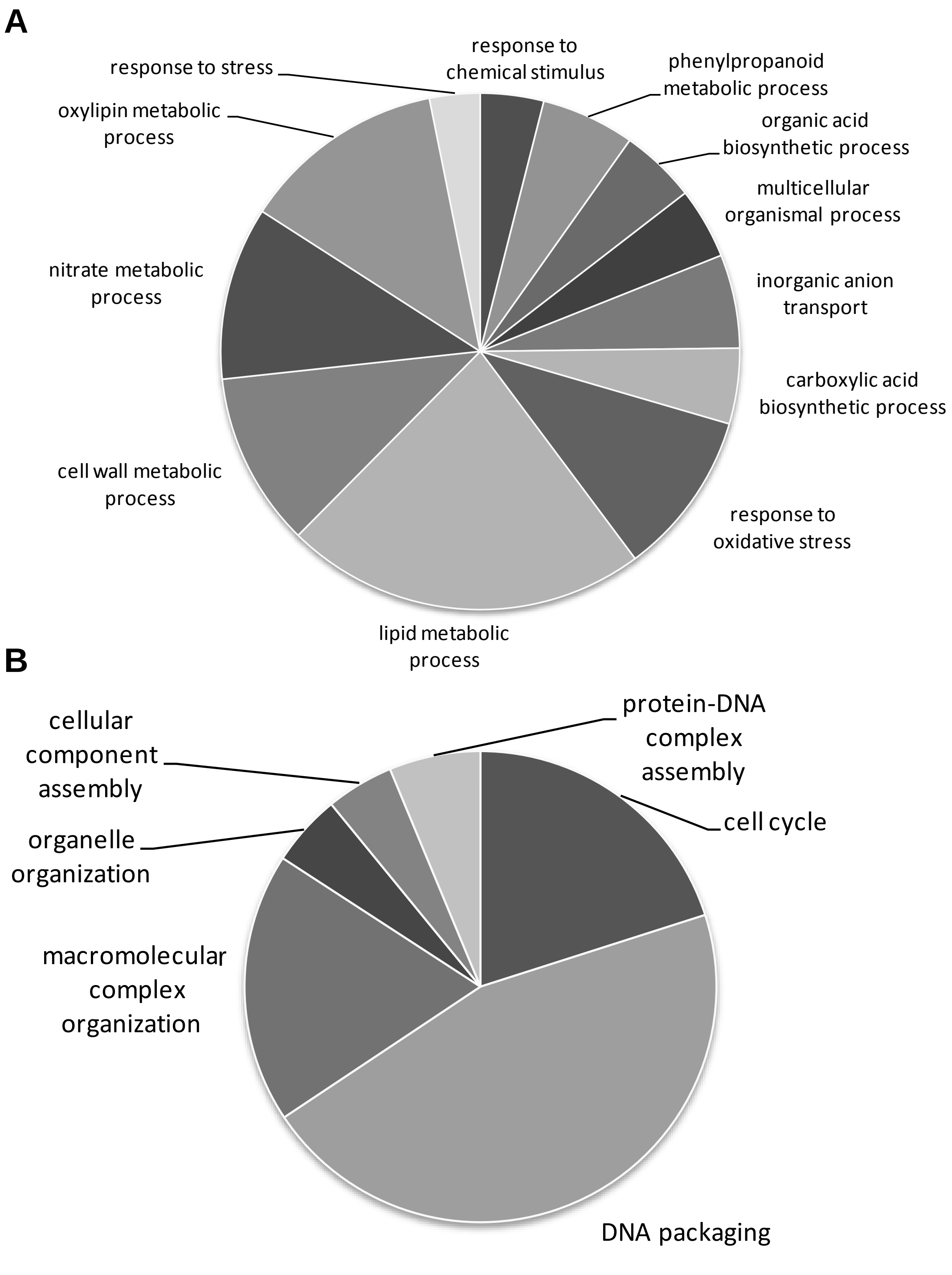

Figure S2. Function analysis of genes differentially expressed in S1587-17 after 1 day of treatment in acid soil. A: Up-regulated; B: Down-regulated. All the genes that didn't present Gene Onthologies were removed from the analysis.

A

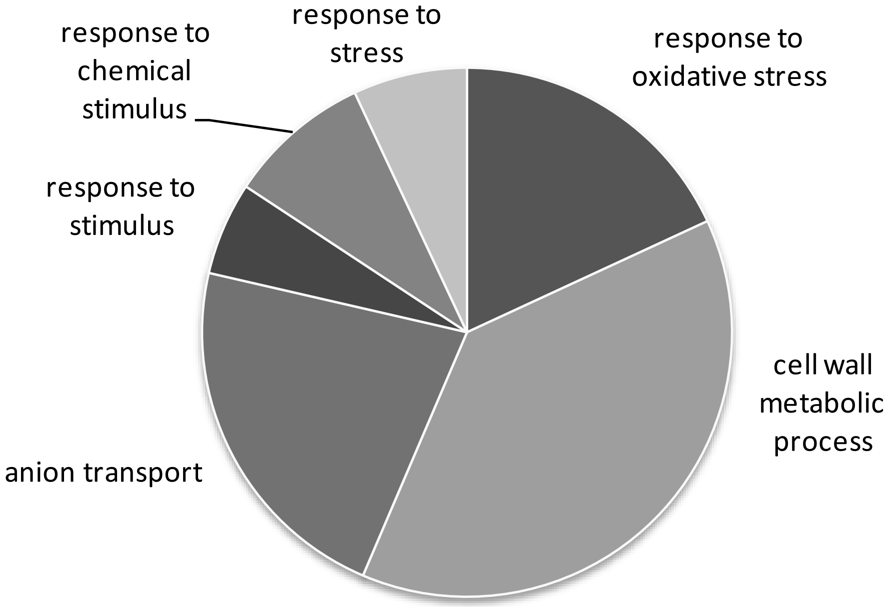

B

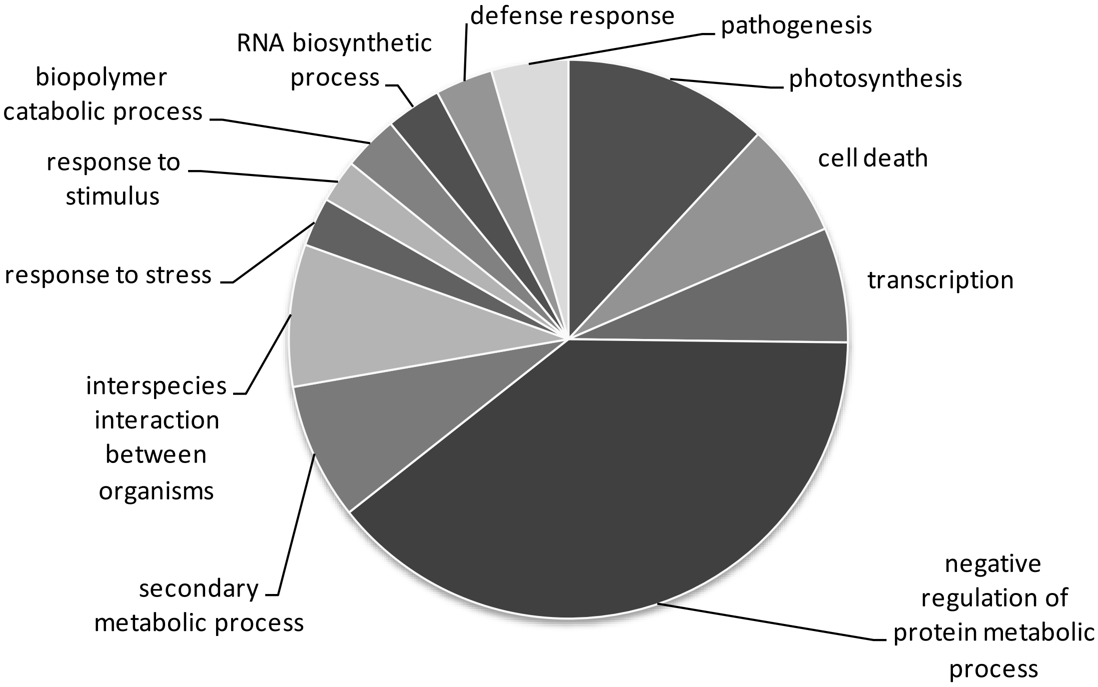

Figure S3. Function analysis of genes differentially expressed in S1587-17 after 3 days of treatment in acid soil. A: Up-regulated; B: Down-regulated. All the genes that didn't present Gene Onthologies were removed from the analysis.
